# Open and arthroscopic deepening trochleoplasty improves post‐operative outcomes: A systematic review of the literature reveals lack of comparability between techniques

**DOI:** 10.1002/ksa.12647

**Published:** 2025-03-17

**Authors:** Signe Høj, Johanne Kofoed Lundegaard, Lars Blønd, Peter Lavard, Anke Simone Rechter, Christian Dippmann, Kristoffer W. Barfod

**Affiliations:** ^1^ Section of Sports Traumatology, Department of Orthopedic Surgery Copenhagen University Hospital Bispebjerg Copenhagen Denmark; ^2^ The Faculty of Health and Medical Sciences University of Copenhagen Copenhagen Denmark; ^3^ Orthopedic Department Zealand University Hospital Køge Denmark; ^4^ Aleris Hospital Copenhagen Denmark

**Keywords:** arthroscopy, patellar dislocation, patellar instability, trochlear dysplasia, trochleoplasty, systematic review

## Abstract

**Purpose:**

Deepening trochleoplasty improves outcomes in patients with trochlear dysplasia. The aim of this systematic review was to present the outcomes after open thin‐flap, open thick‐flap and arthroscopic deepening trochleoplasty.

**Methods:**

A systematic review was conducted using the PRISMA guidelines. Literature was searched in the PubMed, EMBASE and Cochrane databases on 16 December 2024. All studies from the inception of the databases to the date of the search were included in the search. Studies were included if they examined patients with patellar instability and trochlear dysplasia treated with either open or arthroscopic deepening trochleoplasty and reported pre‐ and post‐operative outcomes. Two independent reviewers screened titles and abstracts, reviewed the full text and performed the quality assessment.

**Results:**

A total of 32 studies, consisting of 1435 trochleoplasty cases in 1310 patients, were included. Of the included studies, 21 concerned open thin‐flap trochleoplasty, 8 concerned open thick‐flap trochleoplasty and 3 concerned arthroscopic trochleoplasty. The most used patient‐reported outcome measures were the Kujala score, International Knee Documentation Committee score, visual analogue scale pain score, Lysholm Knee Score and Tegner Activity Scale; and the most reported radiological outcome measures were trochlear sulcus angle, trochlear bump, trochlear depth, tibial tubercle‐trochlear groove distance, Caton–Deschamps index and patellar tilt. All three trochleoplasty techniques lead to improvements in post‐operative outcome measures.

**Conclusion:**

Deepening trochleoplasty improves post‐operative outcome using both open thin‐flap, open thick‐flap and arthroscopic deepening technique. Comparison between the techniques is challenging due to low methodological quality of studies. Further research is needed to document treatment effect and improve patient outcome.

**Level of Evidence:**

Level IV.

**Abbreviations**
CDI: Caton–Deschamps index
IKDC: International Knee Documentation Committee
LTI: lateral trochlear inclination
MINORS: Methodological Index for Non‐Randomized Studies
MPFL: medial patellofemoral ligament
PRISMA: Preferred Reporting Items for Systematic Reviews and Meta‐Analyses
PROM: patient‐reported outcome measure
TT‐TG: tibial tubercle‐trochlear groove
VAS: visual analogue scale

## INTRODUCTION

Patellar instability, a disabling condition particularly affecting young individuals, is commonly associated with anatomical risk factors such as trochlear dysplasia, patella alta, excessive tibial tubercle‐trochlear groove (TT‐TG) distance, and malrotation [16]. Patellar instability is more prevalent in females than males, potentially due to a higher prevalence of these risk factors [23]. Trochlear dysplasia is considered the primary contributor, characterized by a shallow, flat or convex femoral trochlear groove and ranges from subtle to severe [1, 16].

Surgical treatments include medial patellofemoral ligament (MPFL) reconstruction, tibial tubercle osteotomy, derotational osteotomy, and trochleoplasty. Trochleoplasty corrects severe trochlear dysplasia by reshaping the trochlear groove to improve patellar tracking. The most common approach is the deepening trochleoplasty, which removes subchondral bone to create a deeper groove, using either a thin or thick osteochondral flap [37].

The thick‐flap technique utilizes an osteochondral shell with ≥5 mm of subchondral bone, requiring osteotomization of the cartilage, with fixation using screws or staples [13, 15]. In contrast, the thin‐flap technique uses a more pliable 1–2 mm shell, eliminating the need for cartilage osteotomization and allowing fixation with Vicryl, suture anchors or compression tacks [4]. The thin‐flap technique can be performed arthroscopically through suprapatellar portals, involving small incisions, cartilage release, bone removal and flap re‐fixation with suture anchors [7].

The open traditional technique has shown good results but requires extensive surgical exposure. Arthroscopic trochleoplasty, aims to reduce surgical trauma and promote faster recovery. While previous reviews have compared thin‐ and thick‐flap techniques, data on open versus arthroscopic approaches remain limited. Given the widespread adoption of minimally invasive techniques across surgical specialities, it is important to assess whether arthroscopic trochleoplasty achieves comparable outcomes.

The aim of this systematic review was to present the outcomes after deepening trochleoplasty using the open thin‐flap, the open thick‐flap and the arthroscopic techniques.

## MATERIALS AND METHODS

A systematic review was conducted following the Preferred Reporting Items for Systematic Reviews and Meta‐Analyses (PRISMA) guidelines, using a PRISMA checklist [39]. The protocol was prospectively registered in PROSPERO (CRD42024525870).

### Literature search and study selection

A literature search was performed in the PubMed, EMBASE and Cochrane databases on 16 December 2024, using Medical Subject Headings or Emtree terms along with free‐text keywords. The search terms included ‘trochleoplasty’ OR (‘trochlear dysplasia’ AND ‘orthopaedic surgery’). To minimize the number of missed studies, no filters were applied to the search strategy. All studies from the inception of the databases to 16 December 2024, were included in the search, as there was no restriction on the date of publication. Two independent reviewers (S.H. and J.K.L.) screened titles and abstracts screening, followed by a full‐text review of eligible studies. Additionally, reference lists of included studies were manually reviewed for further relevant publications.

Studies were eligible for inclusion if they met the following criteria: (1) studying patients with patellar instability and trochlear dysplasia, (2) studying patients who had undergone either open or arthroscopic deepening trochleoplasty, (3) comparing pre‐ and post‐operative outcomes and (4) studying either patient‐reported outcome measures (PROMs), radiological outcome measures and/or complications related to surgery, including re‐dislocations and re‐operations. Studies reporting on patients undergoing deepening trochleoplasty with concomitant patellar stabilization procedures were included. Exclusion criteria were studies not written in English, cohorts of <10 patients, reviews of the literature, non‐clinical studies, biomechanical, cadaver or animal studies, conference abstracts, case reports, letters, studies where the indication for trochleoplasty was another than patellar instability (e.g., pain) and studies on patellar stabilization procedures in general, without distinguishing the outcomes of the different surgeries from each other (e.g., a cohort where outcomes of isolated MPFL reconstructions, tibial tubercle osteotomies and trochleoplasties were evaluated in the same cohort).

### Data collection

A standardized data extraction protocol was created in Microsoft Excel before the literature search. One reviewer (S.H.) extracted relevant data from each study, including name of author, year of publication, study design, study population (number of knees/number of patients), duration of follow‐up, loss to follow‐up/follow‐up rate, inclusion criteria/surgical indications, exclusion criteria/contraindication, trochleoplasty technique, concomitant patellar stabilization procedures and outcome measures, including all reported PROMs, radiological outcome measures and complications, including re‐dislocations and re‐operations.

### Data analysis

The extracted data revealed significant variability in reported outcomes and statistical descriptors, preventing a meta‐analysis. Instead, tables summarizing the most reported outcome measures were created. An outcome measure was included in the summary tables if both pre‐ and post‐operative measures were available in at least four studies. To compare open and arthroscopic deepening trochleoplasty, changes in outcome measures were calculated by subtracting preoperative values from post‐operative values. Furthermore, re‐dislocation rates were determined by dividing the number of re‐dislocations by the total number of knees that underwent trochleoplasty in studies reporting re‐dislocations.

### Risk of bias assessment

Two reviewers (S.H. and J.K.L.) evaluated the risk of bias using the Methodological Index for Non‐Randomized Studies (MINORS) [42], a validated tool for assessing the quality of non‐randomized studies. MINORS consists of 8 items for non‐comparative studies and 12 items for comparative studies evaluating study quality. Each item is scored 0–2 points, the items were scored 0 if not reported, 1 when reported but inadequate and 2 when reported and adequate. The ideal score was 16 for non‐comparative studies and 24 for comparative studies.

## RESULTS

### Study selection and characteristics

**Figure 1:**
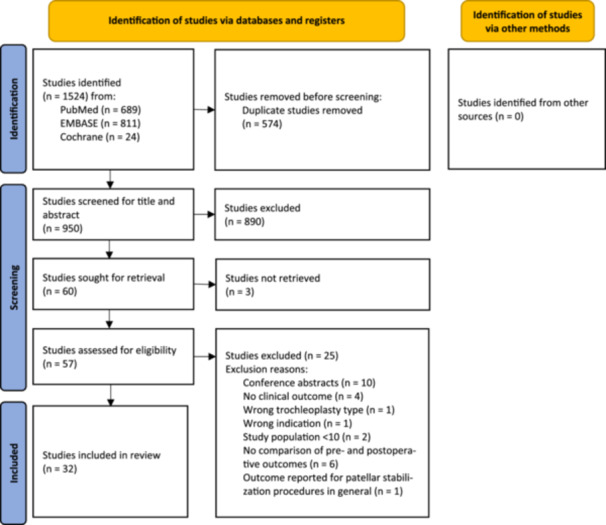
Preferred Reporting Items for Systematic Reviews and Meta‐Analyses (PRISMA) flow diagram.

A total of 1524 studies were identified. After removing duplicates, 950 studies underwent title and abstract screening, and 60 studies were full‐text evaluated (Figure 1). Ultimately, 32 studies [2, 3, 5, 6, 8, 9, 10, 11, 12, 14, 17, 18, 21, 24, 25, 26, 29, 30, 31, 32, 33, 34, 35, 36, 38, 40, 41, 43, 45, 47, 48, 49] comprising 1435 trochleoplasty cases in 1310 patients, were included (Table 1). Of these studies, 21 concerned open thin‐flap trochleoplasty [2, 3, 10, 17, 21, 24, 25, 26, 30, 31, 32, 33, 34, 35, 38, 41, 43, 45, 47, 48, 49], 8 concerned open thick‐flap trochleoplasty [6, 11, 12, 14, 18, 29, 36, 40] and 3 studies concerned arthroscopic trochleoplasty [5, 8, 9]. No studies directly compared any two of the three deepening trochleoplasty techniques. One study was case–control comparing radiological outcomes of open thin‐flap deepening trochleoplasty with controls with no medical history related to the patellofemoral joint [2]. The majority of the studies included concomitant patellar stabilization procedures, but only four studies did not. All studies were at risk of bias according to the MINORS score (Table 2). All non‐comparative studies scored below 16, and the comparative study scored under 24. The primary sources of bias were a non‐blinded assessment of the study end point and no prospective calculation of study size.

**Table 1:** Characteristics of the studies included in this systematic review.

Author (year)	Study design	Knees/patients	Duration of follow‐up	Patellar instability, type of TD			Concomitant procedures							
					MPFLr	TTO	LR	MR	VMOp	VLp	RG	PO	RO	AS
**Open thin‐flap trochleoplasty**														
Balcarek et al. (2019)	Case–control	20/20 (+20 controls)	9 mos.	RPD, Dejour B, C, D	20									
Banke et al. (2014)	Prospective cohort	18/17	30.5 mos.	RPD, Dejour B, C, D	18									
Camathias et al. (2016)	Retrospective cohort	50/44	33 mos.	RPD, Dejour B, C, D										
Dippmann et al. (2024)	Prospective cohort	135/131	5 yrs.	RPD, Dejour B, D	135	70				2			3	3
Falkowski et al. (2017)	Case series	22/22	8.8 mos.	RPD, no TD type reported										
Fucentese et al. (2007)	Case series	17/14	3 yrs.	RPD, radiographic signs of TD			17		17					
Fucentese et al. (2011)	Retrospective cohort	44/38	4 years^a^	RPD, Dejour A, B, C, D			44		44					
Hampton et al. (2020)	Case series	31/27	13–80 mos.^b^	RPD, severe TD (OBC)		3								
Mengis et al. (2022)	Retrospective cohort	112/111	39.2 mos.	Dejour B, D	99	20	71	4					7	
Metcalfe et al. (2017)	Retrospective cohort	199/173	4.43 yrs.	RPD, radiographic signs of TD	1	19	3	174						
Nelitz et al. (2013)	Prospective cohort	26/23	2.5 yrs.	RPD, severe TD (MRI)	26		6	13	1		2			
Nelitz et al. (2018)	Case series	18/18	2.3 yrs.	RPD, Dejour B, C, D	18									
Neumann et al. (2016)	Retrospective cohort	46/42	56.7 mos.^a^	RPD, Dejour A, B, C, D	46				46					
Ng et al. (2023)	Retrospective cohort	58/50	36.8 mos.	RPD, Dejour B, C, D	54	27								
Orfanos et al. (2022)	Retrospective cohort	51/48	21.6 mos.	RPD, severe TD (OBC)		19		51						
Schöttle et al. (2005)	Case series	19/16	3 yrs.	FPD/RPD, radiographic signs of TD										
Tan et al. (2024)	Retrospective cohort	21/21	58 mos.	RPD, Dejour B, D	21	21	21							
Utting et al. (2008)	Prospective cohort	42/40	24 mos.	FPD/RPD/HPD, severe TD (MRI)	18	4			19					
von Engelhardt et al. (2017)	Case series	33/30	29 mos.	RPD, Dejour B, D	33									
von Knoch et al. (2006)	Retrospective cohort	45/38	8.3 yrs.	RPD, radiographic signs of TD										
Wind et al. (2019)	Prospective cohort	22/21	5 yrs.^c^	FPD/RPD, Dejour B, D	22	22	17							
**Open thick‐flap trochleoplasty**														
Blanchard et al. (2024)	Retrospective cohort	21/17	64 mos.	RPD, Dejour B, D	21	10								
Carstensen et al. (2019)	Prospective cohort	62/62	32.5 mos.	RPD, Dejour B, D	62	22	33							
Carstensen et al. (2020)	Prospective cohort	44/40	3.6 yrs.	RPD, Dejour B, D	44	17	22							
Dejour et al. (2013)	Retrospective cohort	24/22	66.5 mos.	RPD, Dejour B, D	14	16	6		10			1		
Donell et al. (2006)	Prospective cohort	17/15	3 yrs.	RPD, radiographic signs of TD		9	17	16						
McNamara et al. (2015)	Retrospective cohort	107/90	6 yrs.	FPD/RPD/HPD, Dejour B, C, D	14	11	28							
Ntagiopoulos et al. (2013)	Retrospective cohort	31/27	7 yrs.	RPD, Dejour B, D	5	37	21		26					
Rouanet et al. (2015)	Retrospective cohort	34/34	15.3 yrs.	FPD/RPD, Dejour A, B, C, D		10								
**Arthroscopic trochleoplasty**														
Bin Zainuddin et al. (2024)	Retrospective cohort	13/13	18 mos.^c^	RPD, Dejour type D	13									
Blønd et al. (2014)	Prospective cohort	37/31	29 mos.^a^	RPD, Dejour type B, C, D	37									
Blønd et al. (2023)	Retrospective cohort	16/15	63.6 mos.	RPD, LTI angle <11°	16									

*Note*: Duration of follow‐up is expressed as mean, unless stated otherwise.

Abbreviations: AS, arthroscopy; FPD, first‐time patellar dislocation; HPD, habitual patellar dislocation; LR, lateral retinaculum release/lengthening; LTI, lateral trochlear inclination; mos., months; MPFLa, medial patellofemoral ligament advancement; MPFLr, medial patellofemoral ligament reconstruction; MR, medial reefing; MRI, magnetic resonance imaging; OBC, Oswestry–Bristol Classification; PO, patellar osteotomy; RG Roux–Goldthwait procedure; RO, realignment osteotomy; RPD, recurrent patellar dislocation; TD, trochlear dysplasia; TTD, tibial tuberosity distalization; TTM, tibial tuberosity medialization; TTO, tibial tuberosity osteotomy; VLp, vastus lateralis plasty; VMOp, vastus medialis obliquus plasty; yrs., years.

a Median.

^b^ Range.

^c^ Minimum.

**Table 2:** Methodological items for non‐randomized studies (MINORS) risk of bias assessment tool.

Author (year)	Clearly stated aim	Inclusion of consecutive patients	Prospective data collection	End points appropriate to study aim	Unbiased assessment of study end point	Follow‐up period appropriate to study aim	<5% lost to follow‐up	Prospective calculation of study size	Adequate control group	Contemporary groups	Baseline equivalence of groups	Adequate statistical analyses	Total score
**Open thin‐flap trochleoplasty**													
Balcarek et al. (2019)	2	2	0	2	1	2	0	0	2	2	2	2	17/24
Banke et al. (2014)	2	2	2	2	1	2	2	2	NA	NA	NA	NA	15/16
Camathias et al. (2016)	2	1	2	2	0	2	2	2	NA	NA	NA	NA	13/16
Dippmann et al. (2024)	2	2	2	2	0	2	1	0	NA	NA	NA	NA	11/16
Falkowski et al. (2017)	2	1	1	2	1	2	2	0	NA	NA	NA	NA	11/16
Fucentese et al. (2007)	2	0	0	2	0	2	0	0	NA	NA	NA	NA	6/16
Fucentese et al. (2011)	2	1	1	2	0	2	1	0	NA	NA	NA	NA	9/16
Hampton et al. (2020)	1	2	0	2	0	2	0	0	NA	NA	NA	NA	7/16
Mengis et al. (2022)	2	1	1	2	0	2	1	0	NA	NA	NA	NA	9/16
Metcalfe et al. (2017)	2	2	1	2	0	2	1	0	NA	NA	NA	NA	10/16
Nelitz et al. (2013)	1	2	2	2	0	2	2	0	NA	NA	NA	NA	11/16
Nelitz et al. (2018)	2	2	0	2	0	2	2	0	NA	NA	NA	NA	10/16
Neumann et al. (2016)	2	2	1	2	0	2	1	0	NA	NA	NA	NA	10/16
Ng et al. (2023)	2	1	1	2	0	2	1	0	NA	NA	NA	NA	9/16
Orfanos et al. (2022)	2	1	1	2	1	2	0	0	NA	NA	NA	NA	5/16
Schöttle et al. (2005)	1	1	0	2	0	2	2	0	NA	NA	NA	NA	8/16
Tan et al. (2024)	2	1	1	2	1	2	0	0	NA	NA	NA	NA	9/16
Utting et al. (2008)	1	2	2	2	0	2	2	0	NA	NA	NA	NA	11/16
von Engelhardt et al. (2017)	1	1	0	2	0	2	0	0	NA	NA	NA	NA	6/16
von Knoch et al. (2006)	1	2	1	2	0	2	1	0	NA	NA	NA	NA	9/16
Wind et al. (2019)	2	2	2	2	0	2	2	0	NA	NA	NA	NA	12/16
**Open thick‐flap trochleoplasty**													
Blanchard et al. (2024)	2	1	1	2	0	2	0	2	NA	NA	NA	NA	10/16
Carstensen et al. (2019)	2	1	2	2	0	2	1	0	NA	NA	NA	NA	10/16
Carstensen et al. (2020)	2	1	2	2	0	2	1	0	NA	NA	NA	NA	10/16
Dejour et al. (2013)	2	1	1	2	0	2	2	0	NA	NA	NA	NA	10/16
Donell et al. (2006)	1	2	2	2	0	2	0	0	NA	NA	NA	NA	9/16
McNamara et al. (2015)	2	1	1	2	0	2	0	0	NA	NA	NA	NA	8/16
Ntagiopoulos et al. (2013)	2	1	1	2	0	2	2	0	NA	NA	NA	NA	10/16
Rouanet et al. (2015)	2	2	1	2	0	2	1	0	NA	NA	NA	NA	10/16
**Arthroscopic trochleoplasty**													
Bin Zainuddin et al. (2024)	2	1	1	2	1	2	2	0	NA	NA	NA	NA	11/16
Blønd et al. (2014)	2	2	2	2	0	2	2	0	NA	NA	NA	NA	12/16
Blønd et al. (2023)	2	1	1	2	0	2	0	0	NA	NA	NA	NA	8/16

*Note*: Items are scored with 0 (not reported), 1 (reported but inadequate) or 2 (reported and adequate). The ideal score is 16 for non‐comparative studies and 24 for comparative studies.

Abbreviation: NA, not applicable.

### Patient‐reported outcome measures

Of the 32 included studies, 27 reported both pre‐ and post‐operative PROMs (Table 3). A total of 16 different PROMs were used, but only five were reported in at least four studies (Table 3). The most often used PROMs were the *Kujala score* (26 studies) [3, 5, 6, 8, 9, 10, 12, 14, 17, 18, 21, 25, 26, 29, 31, 32, 33, 34, 36, 38, 40, 41, 43, 45, 47, 49], *the International Knee Documentation Committee* (IKDC) score (12 studies) [3, 6, 12, 14, 17, 31, 32, 36, 38, 45, 47, 49], the *Visual Analogue Scale* (VAS) pain score (6 studies) [3, 30, 32, 33, 47, 49], *Lysholm Knee Score* (six studies) [5, 10, 17, 21, 45, 47] and the *Tegner Activity Scale* (7 studies) [3, 5, 8, 29, 30, 32, 33]. All studies found significant differences between pre‐ and post‐operative Kujala, IKDC, VAS pain and Lysholm Knee scores. One study from each trochleoplasty technique found significant differences between the pre‐ and post‐operative Tegner Activity Scale [3, 8, 29].

**Table 3:** Pre‐ and post‐operative scores of patient‐reported outcome measures reported in four or more of the included studies.

Author (year)	Kujala score (0–100)				IKDC score (0–100)				VAS pain score (0–10)				Lysholm Knee Score				Tegner Activity Scale (0–10)			
	Pre‐op	Post‐op	*Δ*	*p*	Pre‐op	Post‐op	*Δ*	*p*	Pre‐op	Post‐op	Δ	*p*	Pre‐op	Post‐op	*Δ*	*p*	Pre‐op	Post‐op	*Δ*	*p*
**Open thin‐flap trochleoplasty**																				
Banke et al. (2014)	51.1 ± 22.9	87.9 ± 12.9	+36.8	*p* < 0.01	49.5 ± 20.7	80.2 ± 13.7	+30.7	*p* < 0.01	5.6 ± 2.8	2.5 ± 1.7	−3.1	*p* < 0.01					2 (0–4)^a^	6 (3–8)^a^	+4	*p* < 0.01
Camathias et al. (2016)	71 ± 1.1	92 ± 0.8	+20.8	*p* < 0.01									71 ± 1.6	95 ± 0.7	+24	*p* < 0.01				
Dippmann et al. (2024)	60.7 (13–92)	79.8 (27–100)	+19.1	*p* < 0.05	49.9 (21–89)	70.2 (8–100)	+20.3	*p* < 0.05					61 (31–95)	82 (26–100)	+21	*p* < 0.05				
Falkowski et al. (2017)	71.38	90.48	+19.1	*p* < 0.01									73.71	94.48	+22.7	*p* < 0.01				
Fucentese et al. (2011)	68 (29–84)^a^	90 (42–100)^a^	+22	*p* < 0.01																
Hampton et al. (2020)	53.9 (26–79)	91.2 (88.6–100)	+37.3	*p* < 0.01																
Mengis et al. (2022)									3.5 ± 2.8	1.1 ± 1.7	−2.4	*p* < 0.01					4.5 ± 2.4	4.7 ± 1.6	+0.2	*p* = 0.37
Metcalfe et al. (2017)	51.5 (26.5)^a,b^	82.5 (30.5)^a,b^	+31	*p* < 0.01	44.3 (25.3)^a,b^	71.3 (39.1)	+27	*p* < 0.01												
Nelitz et al. (2013)	79 (21–100)^a^	96 (74–100)^a^	+17	*p* < 0.01	74 (32–95)^a^	90 (65–98)^a^	+16	*p* < 0.01	3 (1–7)^a^	1 (0–5)^a^	−2	*p* < 0.01					5.5 (2–8)^a^	5 (2–8)^a^	−0.5	*p* = 0.06
Nelitz et al. (2018)	67 (54–75)^a^	89.5 (78–96)^a^	+22.5	*p* < 0.01					5 (3–7)^a^	1 (0–3)^a^	−2	*p* < 0.01					5 (3–8)^a^	5 (3–8)^a^	0	*p* = 0.20
Neumann et al. (2016)	62 (9–96)^a^	88 (47–100)^a^	+26	*p* < 0.01																
Orfanos et al. (2022)	58.1 ± 14.9	77.9 ± 17.3	+19.8	*p* < 0.01	40.5 ± 14.2	69.5 ± 22.8	+29	*p* < 0.01												
Schöttle et al. (2005)	56.1 (27–69)	80.0 (43–99)	+23.9	*p* < 0.01																
Tan et al. (2024)	36.1 ± 12.9	93.1 ± 3.6	+30	*p* < 0.01																
Utting et al. (2008)	62 (29–92)	76 (26–100)	+14	*p* < 0.01	54 (26–89)	72 (23–100)	+18	*p* < 0.01					57 (25–91)	78 (30–100)	+21	*p* < 0.01				
von Engelhardt et al. (2017)	64 ± 16	94 ± 9	+30	*p* < 0.01	58 ± 11	85 ± 12	+27	*p* < 0.01	4.8 ± 2.0	1.3 ± 3.4	−3.5	*p* < 0.01	63 ± 17	95 ± 6	+32	*p* < 0.01				
Wind et al. (2019)	44 (30–85)^a^	71 (32–93)^a^	+27	*p* < 0.01	43 ± 13	64 ± 16	+21	*p* < 0.01	58 (0–87)^a,d^	18 (2–73)^a,d^	−40	*p* < 0.01								
**Open thick‐flap trochleoplasty**																				
Blanchard et al. (2024)	55 (49–72)^a,b^	95 (92.3–100)^a,b^	+40	*p* < 0.01	57 (49–69)^a,b^	91 (80–99)^a,b^	+34	*p* < 0.01												
Carstensen et al. (2020)	56.4 ± 19.2	86.5 ± 14.2	+30.1	*p* < 0.01	50.8 ± 19.5	79.1 ± 19.5	+28.3	*p* < 0.01												
Dejour et al. (2013)	44.8 ± 15.0	81.7 ± 13.9	+36.9	*p* < 0.01	51.4 ± 21.8	76.7 ± 13.0	+25.3	*p* < 0.01												
Donell et al. (2006)	48 (13–75)	75 (51–98)	+27	*p* < 0.05																
McNamara et al. (2015)	63 (47–75)^a,b^	84 (73–92)^a,^ ^b^	+21	*p* < 0.05													3.3 ± 2.7	4.3 ± 2.1	+1	*p* < 0.01
Ntagiopoulos et al. (2013)	59 (28–81)	87 (49–100)	+28	*p* < 0.01	51.2 ± 22.9	82.5 ± 17.9	+31.3	*p* < 0.01												
Rouanet et al. (2015)	55 (13–75)	76 (51–94)	+21	*p* < 0.01																
**Arthroscopic trochleoplasty**																				
Bin Zainuddin et al. (2024)	50.3 ± 12.0	95.4 ± 4.8	+45.1	*p* < 0.01									68.2 ± 10.3	98.7 ± 2.1	+30.5	*p* < 0.01	6.5 ± 1.5^c^	6.2 ± 1.2	−0.3	NS
Blønd et al. (2014)	64 (12–90)^a^	95 (47–100)^a^	+31	*p* < 0.01													4 (1–6)^a^	6 (4–9)^a^	+2	*p* < 0.01
Blønd et al. (2023)	61.5 (30–84)^a^	88.0 (42–97)^a^	+26.5	*p* < 0.01																

*Note*: Depending on the available data, the values are expressed as mean ± SD or ^a^median (range).

Abbreviations: IKDC, International Knee Documentation Committee; NS, not significant; VAS, visual analogue scale.

^b^ Interquartile range.

^c^ Premorbid score.

^d^ VAS pain score (0–100).

### Radiological measures

Of the 32 included studies, 17 reported both pre‐ and post‐operative radiological outcome measures (Table 4). A total of 28 different radiological outcome measures were used, but only six were reported in at least four studies (Table 4). The most often used radiological measures were *trochlear sulcus angle* (seven studies) [3, 12, 14, 24, 36, 43, 49], *trochlear bump* (four studies) [18, 29, 48, 49], *trochlear depth* (seven studies) [9, 24, 34, 41, 43, 48, 49], *TT‐TG distance* (eight studies) [3, 9, 14, 21, 25, 36, 41, 43], *Caton–Deschamps index* (CDI) (nine studies) [3, 14, 25, 29, 34, 36, 40, 41, 43] and *lateral patellar inclination angle* (nine studies) [2, 3, 9, 14, 24, 25, 36, 41, 43].

**Table 4:** Pre‐ and post‐operative radiological measures reported in four or more of the included studies.

Author (year)	Trochlear morphology												Tibial tubercle‐trochlear groove (TT‐TG) distance				Patellar height				Patellar tilt			
	Trochlear sulcus angle (°)				Trochlear bump (mm)				Trochlear depth (mm)				TT‐TG distance (mm)				CDI				Lateral patellar inclination (°)			
	Pre‐op	Post‐op	*Δ*	*p*	Pre‐op	Post‐op	*Δ*	*p*	Pre‐op	Post‐op	*Δ*	*p*	Pre‐op	Post‐op	*Δ*	*p*	Pre‐op	Post‐op	*Δ*	*p*	Pre‐op	Post‐op	*Δ*	*p*
**Open thin‐flap trochleoplasty**																								
Balcarek et al. (2019)																					25.8 (8.1–43.0)	13.9 (8.1–22.1)	−11.9	*p* < 0.01
Banke et al. (2014)	154.0 ± 13.5	143.3 ± 9.4	−10.7	*p* < 0.01									16.2 ± 5.7	10.7 ± 5.3	−5.5	*p* < 0.01	1.09 ± 0.16	1.0 ± 0.17	−0.09	*p* < 0.01	24.2 ± 7.5	15.8 ± 5.3	−8.4	*p* < 0.01
Falkowski et al. (2017)													14 (5–25)	10 (5–17)	−4	*p* < 0.01								
Fucentese et al. (2007)	172.1 (150–190)	133 (110–150)	−39.1						0 (−5 to 4)	5.9 (1–11)	+5.9										21.9 (5–35)	7.8 (1–20)	−14.1	
Fucentese et al. (2011)													17 (11–30)^a^	13 (0–21)^a^	−4	*p* < 0.01	1.0 (0.7–1.5)^a^	0.9 (0.7–1.3)^a^	−0.1	*p* < 0.01	32 (11–60)^a^	13 (−3 to 36)^a^	−19	*p* < 0.01
Neumann et al. (2016)									4.7 (1.3–7.7)^a^	7.3 (4.1–11.5)^a^	+2.6	*p* < 0.01					1.2 (0.8–2.2)^a^	1.0 (0.8–1.3)^a^	−0.2	*p* < 0.01				
Schöttle et al. (2005)									0.6 (−3 to 3)	7 (4–9)	+6.4	*p* < 0.01	20 (10–24)	9.9 (5–15)	−10.1	*p* < 0.01	1.2 (0.9–1.5)	1.1 (0.8–1.3)	−0.1	NS	21.9 (5–35)	7.8 (1–20)	−14.1	*p* < 0.01
Tan et al. (2024)	152.9 ± 9.3	146.1 ± 7.8	−6.8	*p* < 0.01					3.0 ± 1.6	3.8 ± 1.6	+0.8	*p* = 0.04	22.5 ± 3.0	9.6 ± 2.7	−12.9	*p* < 0.01	1.2 ± 0.03	1.0 ± 0.1	−0.2	*p* < 0.01	41.3 ± 15.0	17.2 ± 12.8	−24.1	*p* < 0.01
von Knoch et al. (2006)					3.9 (0–10)	0.4 (−3 to 6)	−3.5	*p* < 0.01	−0.1 (−7 to 5)	5 (0–10)	+5.1	*p* < 0.01												
Wind et al. (2019)	149.9 ± 8.8	137.6 ± 5.7	−12.3	*p* < 0.01	8.1 ± 3.0	−1.6 ± 2.1	−9.7	*p* < 0.01	0.6 (−3.7 to 3.7)^a^	4.3 (2.2–8.7)^a^	+3.7	*p* < 0.01												
**Open thick‐flap trochleoplasty**																								
Carstensen et al. (2020)	146.3 ± 7.1	134.6 ± 9.5	−11.7	*p* < 0.01																				
Dejour et al. (2013)	153 ± 14.7	141 ± 10.2	−12	*p* < 0.01									16.6 ± 7.7	12.6 ± 4.2	−4	*p* < 0.01	1.03 ± 0.28	0.95 ± 0.22	−0.08	NS	31.4 ± 14.3	11.8 ± 8.0	−19.6	*p* < 0.01
Donell et al. (2006)					7.5 (6–11)	0.7 (−1 to 3)	−6.8	*p* < 0.01																
McNamara et al. (2015)					6.55 ± 1.9	0.03 ± 1.2	−6.52	*p* < 0.01									1.10 ± 0.20	0.98 ± 0.18	−0.12	*p* < 0.01				
Ntagiopoulos et al. (2013)	152 ± 16.2	141 ± 8.9	−11	*p* < 0.01									19 ± 4.8	12 ± 5	−7	*p* = 0.01	1.12 ± 0.18	0.97 ± 0.15	−0.15	NS	37 ± 7.8	15 ± 8.7	−22	*p* < 0.01
Rouanet et al. (2015)																	1.16 (0.8–2)	1.12 (0.7–1.2)	−0.04	NS				
**Arthroscopic trochleoplasty**																								
Blønd et al. (2023)									1.73 (0.0–3.0)^a^	4.93 (0.99–7.55)^a^	+3.2	*p* < 0.01	14.3 (5.9–19.5)^a^	12.5 (8.2–20.6)^a^	−1.8	NS					20.7 (16.0–36.4)^a^	14.7 (5.3–24.8)^a^	−6	*p* < 0.01

*Note*: Depending on the available data, the values are expressed as mean ± SD/(range) or ^a^median (range).

Abbreviations: CDI, Caton–Deschamps index; NS, not significant.

#### Trochlear morphology

With the exception of one study that did not calculate statistical significance [24], all studies found significant differences between pre‐ and post‐operative trochlear morphology measures. Measures of trochlear sulcus angles and trochlear bump were only reported in open trochleoplasty studies. Trochlear depths were reported in open thin‐flap studies and in one arthroscopic study.

#### TT‐TG distance

All open thin‐flap and open thick‐flap studies showed significant differences between pre‐ and post‐operative TT‐TG distances. The arthroscopic study found no significant difference between pre‐ and post‐operative TT‐TG distances.

#### Patellar height

CDI was only reported in open trochleoplasty studies. Four open thin‐flap and one open thick‐flap reported significant differences between pre‐ and post‐operative CDI.

#### Patellar tilt

Except for one study that did not calculate statistical significance [24], all studies found significant differences between pre‐ and post‐operative lateral patellar inclination angle.

### Complications following deepening trochleoplasty

Table 5 provides an overview of complications, including re‐dislocations and re‐operations, following deepening trochleoplasty. Of the 32 included studies, 26 reported re‐dislocations [3, 5, 6, 8, 9, 10, 11, 12, 14, 17, 18, 25, 26, 29, 30, 31, 32, 33, 34, 35, 36, 38, 40, 41, 45, 47, 48, 49] and 26 studies reported re‐operations [3, 5, 6, 8, 9, 10, 11, 12, 14, 17, 18, 25, 26, 29, 30, 31, 32, 33, 35, 36, 38, 40, 41, 45, 47, 48, 49].

**Table 5:** Complications following deepening trochleoplasty.

Author (year)	Knees/patients	Re‐dislocations	Re‐operations												Other complications
			MUA	MPFLr	R‐MPFL	re‐MR	LR	HR	AS	TTO	AL	2nd L	RO	AP	
**Open thin‐flap trochleoplasty**															
Banke et al. (2014)	18/17	0 (0%)			1						2				None other than re‐operations were reported
Camathias et al. (2016)	50/44	1 (2%)		1							4				6 persistent J‐signs. 8 persistent positive apprehension tests.
Dippmann et al. (2024)	135/131	3 (2%)		4				9	8	1	24	2		1	No deep vein thrombosis, no confirmed deep joint infection, no trochleoplasty revision. 2 cosmetic scar revision. 1 synovectomy and 6‐weeks antibiotics
Falkowski et al. (2017)	22/22	NR		NR											3 persistent J‐signs. 6 persistent positive apprehension tests.
Fucentese et al. (2011)	44/38	1 (2.3%)		1					3	1					1 post‐operative femoral nerve palsy, 1 wound‐healing problem and 1 regional pain syndrome. No infections, no thromboembolic events, no haematomas
Hampton et al. (2020)	31/27	0 (0%)							1						1 post‐operative stiffness. 1 persistent post‐operative pain
Mengis et al. (2022)	112/111	3 (2.7%)		1				6	1	2	6				None other than re‐operations were reported
Metcalfe et al. (2017)	199/173	16 (8%)	2	9	1			2	4	7	2				1 partial detachment of the cartilage flap. 1 regional pain syndrome. 1 drop foot. No deep vein thromboses, no pulmonary emboli, no infections.
Nelitz et al. (2013)	26/23	0 (0%)							1						1 reduced flexion 6 weeks after surgery. No wound infection, no deep infection
Nelitz et al. (2018)	18/18	0 (0%)									1				1 persistent apprehensions and J‐sign. 2 reduced ROM. No other complications were recorded
Ng et al. (2023)	58/50	3 (5%)	3	1				5	2						No infections, no chondral necrosis, no nonunion
Orfanos et al. (2022)	51/48	0 (0%)	3	1				1							None other than re‐operations were reported
Schöttle et al. (2005)	19/16	0 (0%)		NR											4 persistent apprehensions. 5 tenderness of the medial patellofemoral retinacular area. 2 increased pain post‐operative
Utting et al. (2008)	42/40	1 (2.3%)	1												2 superficial wound infection. No cases of deep infection. 1 anaphylactic reaction after prophylactic antibiotics. No thromboembolic events. No chondrolysis, no nonunion
von Engelhardt et al. (2017)	33/30	0 (0%)									2				No deep or wound infection. No patella fractures. No breakages of the osteochondral flap
von Knoch et al. (2006)	45/38	0 (0%)								1					1 patella baja
Wind et al. (2019)	22/21	0 (0%)	3					2							4 persistent J‐signs
Total	925/847	28 (3.1%)	12	18	2	0	0	25	20	12	41	2	0	1	
**Open thick‐flap trochleoplasty**															
Blanchard et al. (2024)	21/17	1(4.8%)	3					1		1	3		1		None other than re‐operations were reported
Carstensen et al. (2019)	62/62	0 (0%)	11								9				None other than re‐operations were reported
Carstensen et al. (2020)	44/40	2 (4.5%)	8					1	3		6				None other than re‐operations were reported
Dejour et al. (2013)	24/22	0 (0%)						1							No post‐operative stiffness. No major complications
Donell et al. (2006)	17/15	NR				1		11	1		5				6 with crepitus
McNamara et al. (2015)	107/90	0 (0%)		10				2	1		8				1 deep vein thrombosis and 1 pulmonary embolus, 4 superficial wound infections, no deep infections, 4 with crepitus, 1 stiff knee
Ntagiopoulos et al. (2013)	31/27	0 (0%)						2							1 deep venous thrombosis
Rouanet et al. (2015)	34/34	0 (0%)	6							1	2			6	8 (23%) post‐operative stiffness at <90° flexion
Total	340/307	3 (0.9%)	28	10	0	1	0	18	5	2	33	0	1	6	
**Arthroscopic trochleoplasty**															
Bin Zainuddin et al. (2024)	13/13	0 (0%)													No re‐operations, no surgical site infection, no arthrofibrosis,
Blønd et al. (2014)	37/31	0 (0%)					3			2					2 pts with subluxations. No infections, no cartilage flake breakage, no necrosis
Blønd et al. (2023)	16/15	0 (0%)													No re‐operations, no deep vein thrombosis, no infection, no reduced ROM
Total	66/59	0 (0%)	0	0	0	0	3	0	0	2	0	0	0	0	

Abbreviations: 2nd L, second look; AL, arthrolysis; AP, arthroplasty; AS, arthroscopy; HR, hardware‐removal; LR, lateral release; MPFLr, medial patellofemoral ligament reconstruction; MUA, manipulation under anaesthesia; NR, not reported; R‐MPFL, release of tight medial patellofemoral ligament; re‐MR, re‐medial reefing; RO, realignment osteotomy; ROM, range of motion; TTO, tibial tubercle osteotomy.

Re‐dislocation rates were 3.1% (28/903) for open thin‐flap, 0.9% (3/323) for open thick‐flap and 0% (0/66) for arthroscopic trochleoplasty. Notably, 16 of the 28 re‐dislocations in the open thin‐flap group were reported in a single study. No statistical analysis was conducted to compare re‐dislocation rates among the three techniques.

The most common re‐operations reported following open trochleoplasties were arthrolysis and manipulation under anaesthesia to treat stiffness and arthrofibrosis, along with hardware removal, primarily related to concomitant patellar stabilization procedures. In contrast, no cases of arthrolysis were reported following arthroscopic trochleoplasty. The only re‐operations in this group were lateral release and tibial tubercle osteotomy, performed to address post‐operative lateral hyper‐pressure syndrome and subluxations, respectively.

## DISCUSSION

All included studies, regardless of whether they examined open thin‐flap, open thick‐flap or arthroscopic deepening trochleoplasty, concluded that deepening trochleoplasty improved outcomes. However, comparing techniques is challenging due to the low methodological quality of the studies and the wide variation in outcome measures and statistical descriptors. Additionally, differences in surgical techniques and the pathology of the included patient populations further complicate direct comparisons between open and arthroscopic techniques.

The open trochleoplasty group comprised 1369 knees, with 1082 additional procedures beyond MPFL reconstruction. In contrast, the arthroscopic trochleoplasty group comprised only 66 knees, all of which had MPFL reconstruction as the only additional procedure. This disparity suggests that the two groups may not be directly comparable. Given the morphological differences among patients with trochlear dysplasia, individualized treatment approaches may be necessary. Different grades of dysplasia may benefit from different surgical approaches, though this remains an area for further investigation.

### Patient‐reported outcome measures

All studies reporting Kujala, IKDC, VAS pain and Lysholm Knee scores found significant improvements after deepening trochleoplasty. Improvement in Kujala and IKDC scores has also been observed in previous systematic reviews of trochleoplasty [19, 20, 22, 28, 44]. Longo et al. reviewed clinical outcomes of various trochleoplasty techniques (open thin‐flap, open thick‐flap and Goutallier recession), and found significant improvements in post‐operative Kujala scores across all techniques [28]. Similarly, Eikani et al. compared open thin‐flap and open thick‐flap trochleoplasty and found no significant difference in Kujala score improvement between the two techniques [20].

The Tegner Activity Scale was used in studies across all three trochleoplasty techniques, with a comparable range of change scores. Significant improvements in Tegner Activity Scale were not consistently observed; four studies found no significant difference between pre‐ and post‐operative scores. This suggests that while some patients may experience an increase in activity level following deepening trochleoplasty, others may return only to their preoperative level of activity. A recent study by Tarchala et al. reported that 40%–92% of patients were able to return to sports following deepening trochleoplasty [44]. In a review of trochleoplastyoutcomes, Hiemstra et al. noted that return‐to‐sport reporting is limited, but available data suggest that patients generally improve their activity levels after trochleoplasty [27].

### Radiological measurements

Direct comparison of radiological outcomes is difficult due to inconsistencies in measurement techniques across institutions. Standardized protocols for radiological assessment are needed but are rarely provided in the studies, limiting the ability to draw definitive conclusions. Despite this, both open and arthroscopic trochleoplasty improved radiological measures.

Studies on open thin‐flap and open thick‐flap trochleoplasty reported significantly smaller trochlear bumps and improved trochlear sulcus angles post‐operatively, while these measures were not reported in the arthroscopic studies. A systematic review by Dwi Damayanthi et al. comparing thin‐flap and thick‐flap trochleoplasty found no significant differences in post‐operative improvements of trochlear sulcus angle between the two techniques [19].

TT‐TG distances significantly decreased after open thin‐flap and open thick‐flap trochleoplasty, whereas no improvement was observed following arthroscopic trochleoplasty. Notably, the post‐operative median TT‐TG distance in the arthroscopic group was similar to that of the open technique groups. Blønd et al. found no significant difference between pre‐ and post‐operative TT‐TG distances [9], likely because their study included patients with a lower median preoperative TT‐TG distance compared to the open technique groups, making direct comparison difficult.

A wide variety of radiological outcome measures were used across studies, with some becoming outdated as the field evolves. For instance, the lateral trochlear inclination (LTI) angle is considered an important parameter for assessing trochlear dysplasia. However, the LTI angle was not included in this review since only two of the 32 included studies reported both pre‐ and post‐operative LTI angles [9].

### Complications

Open thin‐flap, open thick‐flap and arthroscopic trochleoplasty all showed low re‐dislocations rates with 25/835 (3.0%), 2/302 (0.7%) and 0/66 (0%), respectively. However, the authors are aware of at least two cases of re‐dislocations in patients who underwent arthroscopic trochleoplasty and suspect that the re‐dislocation rates reported in the included retrospective cohort studies may underestimate the actual rate.

A systematic review and meta‐analysis by van Sambeeck et al. evaluated complication rates after trochleoplasty [46]. Their meta‐analysis of 20 studies found re‐dislocation rates of 4% for thin‐flap trochleoplasties and 2% for thick‐flap trochleoplasties, though the difference was not statistically significant.

Arthrolysis and manipulation under anaesthesia to treat stiffness and arthrofibrosis were common re‐operations in the open trochleoplasty groups. Eikani et al., also found that arthrofibrosis was the most frequently reported complication in both thin‐flap and thick‐flap trochleoplasty, occurring in 2.4% of thin‐flap and 4.8% of thick‐flap cases [20]. Carstensen et al. reported that 11 out of 62 patients who underwent open thick‐flip trochleoplasty required manipulation under anaesthesia within three months post‐operatively due to arthrofibrosis [11]. In contrast, no cases of arthrolysis or manipulation under anaesthesia were reported after arthroscopic trochleoplasty.

### Limitations

This review is limited by the methodological quality of the included studies. A wide variation in reported outcomes and statistical descriptors precluded meta‐analysis. Instead, change scores were calculated to summarize differences in outcome measures, but these remain difficult to compare, as some are based on mean values and others on median values. Additionally, differences in the severity of trochlear dysplasia and the extent of concomitant pathology further complicate comparisons.

Furthermore, most PROMs used in the studies have not been specifically validated for patellar instability patients. The Banff Patellofemoral Instability Instrument 2.0 is validated for this patient group, but it was reported pre‐ and post‐operatively in only two of the included studies [9, 30]. The overall low methodological quality of the studies makes comparing deepening trochleoplasty techniques challenging. High‐quality comparative studies are urgently needed. In December 2024, a retrospective study comparing open and arthroscopic trochleoplasty was published but was not included in this review due to missing preoperative values. The study found comparable outcomes between the two techniques but emphasized the need for prospective research with long‐term follow‐up.

## CONCLUSION

Deepening trochleoplasty improves post‐operative outcomes across open thin‐flap, open thick‐flap, and arthroscopic deepening techniques. Comparison between the techniques is challenging due to the low methodological quality of studies. Populations vary in severity of trochlear dysplasia and concomitant pathology across patient cohorts. Both patient‐reported and radiological outcomes lack validation and standardization, limiting their reliability. Further research is essential to better document treatment effects and optimize patient outcomes in the surgical management of patellar instability.

## AUTHOR CONTRIBUTIONS

Signe Høj and Kristoffer W. Barfod conceived and designed the study and developed the search strategy. Signe Høj performed the literature search. Signe Høj and Johanne Kofoed Lundegaard screened the titles and abstracts for relevance, conducted a full‐text review and performed a quality assessment of included studies. Signe Høj extracted data and conducted the data analysis and interpretation. Signe Høj and Kristoffer W. Barfod wrote the initial draft of the manuscript. All authors critically revised the manuscript. Kristoffer W. Barfod supervised the whole process. All authors reviewed and approved the final version of the manuscript.

## CONFLICTS OF INTEREST STATEMENT

Lars Blønd declares a non‐financial conflict of interest as he is a co‐developer of the arthroscopic trochleoplasty and believes in the superiority of this procedure. Peter Lavard, Anke Simone Rechter and Christian Dippmann declare a non‐financial conflict of interest as they perform and believe in the superiority of the open thin‐flap trochleoplasty. The remaining authors declare no conflicts of interest.

## ETHICS STATEMENT

No ethical approval was required for this systematic review.

## Supporting information

Supporting information.

Supporting information.

## Data Availability

All data extracted and analyzed for this systematic review are included in the article and its Supporting Information. The full search strategy and extraction spreadsheet is available in Appendices S1 and S2. No primary data were collected for this review; all information was derived from published studies that are available through their respective journals or databases.
