# Supplementary material for: Open and arthroscopic deepening trochleoplasty improves post‐operative outcomes: A systematic review of the literature reveals lack of comparability between techniques
Source: Knee Surg Sports Traumatol Arthrosc. 2025 Mar 17;34(1):34–51. doi: 10.1002/ksa.12647 (PMC12747630; doi:10.1002/ksa.12647)
Supplement: Supplementary file 1 — Supporting information. [file KSA-34-34-s002.docx]

| **PubMed** | **Aspect 1 (#1)** | **Aspect 2 (#2)** | **Aspect 3 (#3)** | **(#4)** | **(#5)** |
| --- | --- | --- | --- | --- | --- |
|  | **Surgical procedure** | **Disorder** | **Type of intervention** | **#2 AND #3** | **#1 OR #4** |
| **Controlled vocabularies**  (MeSH terms) |  |  | "Orthopedic Procedures"[Mesh] |  |  |
| **Free text terms** | trochleoplast* OR trochleaplast* | "trochlear dysplas*" OR “trochlear groove dysplas*" | "orthopedic procedur*" or "orthopaedic procedur*" or "orthopedic surger*" or "orthopaedic surger*" or "surger*" or "orthopedic surgical procedur*" or "orthopaedic surgical procedur*" or "arthroscopic surgery" or "open surgery" or "joint surgery" or "knee surgery" or surgery |  |  |
| **Results** | 273 | 723 | 5,970,127 | 561 | 689 |

The search was conducted on 16 December 2024.

The search was conducted on 16 December 2024.

| **EMBASE** | **Aspect 1 (#1)** | **Aspect 2 (#2)** | **Aspect 3 (#3)** | **(#4)** | **(#5)** |
| --- | --- | --- | --- | --- | --- |
|  | **Surgical procedure** | **Disorder** | **Type of intervention** | **#2 AND #3** | **#1 OR #4** |
| **Controlled vocabularies**  (Emtree terms) |  |  | arthroscopic surgery/ or open surgery/ or joint surgery/ or knee surgery/ or orthopedic surgery/ or surgery/ |  |  |
| **Free text terms** | trochleoplast* OR trochleaplast* | "trochlear dysplas*" OR “trochlear groove dysplas*" | "orthopedic procedur*" or "orthopaedic procedur*" or "orthopedic surger*" or "orthopaedic surger*" or "surger*" or "orthopedic surgical procedur*" or "orthopaedic surgical procedur*" or "arthroscopic surgery" or "open surgery" or "joint surgery" or "knee surgery" or surgery |  |  |
| **Results** | 399 | 956 | 4,631,793 | 622 | 811 |
